# Investigation into the Storage-Induced Oxidation Mechanism of Prussian Blue Analogues

**DOI:** 10.3390/ma19142967

**Published:** 2026-07-09

**Authors:** Jieyuan Wang, Jun Zheng, Kai Zhang, Junwei Li, Zhilu Yang, Yueying Lin, Fang Lin, Zijuan Zhou, Sumuqin Zhao, Ming Zhang, Zhongrong Shen

**Affiliations:** 1 CAS Key Laboratory of Design and Assembly of Functional Nanostructures, Fujian Key Laboratory of Nanomaterials, Fujian Institute of Research on the Structure of Matter, Chinese Academy of Sciences, Fuzhou 350002, China; wjy18934316461@163.com (J.W.); xmzhangkai@fjirsm.ac.cn (K.Z.); lijunwv1@163.com (J.L.); yangzhilu1999@163.com (Z.Y.); linyueing123@163.com (Y.L.); lf1979617025@163.com (F.L.); a949682729@163.com (Z.Z.); smqzhao@outlook.com (S.Z.); mingzhang@fjirsm.ac.cn (M.Z.); 2 College of Chemistry and Materials Science, Fujian Normal University, Fuzhou 350007, China; 3 Xiamen Key Laboratory of Rare Earth Photoelectric Functional Materials, Xiamen Institute of Rare Earth Materials, Haixi Institutes, Chinese Academy of Sciences, Xiamen 361021, China; 4 Fujian College, University of Chinese Academy of Sciences, Fuzhou 350007, China; 5 University of Chinese Academy of Sciences, Beijing 101408, China

**Keywords:** Prussian blue analogues, oxidation mechanism, single-iron source, sodium-ion batteries

## Abstract

**Highlights:**

- Oxidative degradation of PBAs requires both water and oxygen. Dry oxygen alone does not cause decomposition, but oxygen accelerates it in humid conditions.
- Water triggers degradation by driving Na+ deintercalation, transforming the structure from monoclinic to cubic, and forming Na4Fe(CN)6·10H2O impurities.
- KPB remains stable in humid air, repelling water molecules due to the larger K+ radius via steric hindrance.
- Low-spin iron is more sensitive to moisture; its preferential deactivation is the key cause of the decline in high-rate performance and cycling stability.
- A synergistic mechanism model of “water-driven degradation and oxygen-accelerated process” has been established.

**Abstract:**

This study reports the synthesis of low-defect Prussian blue analogues (PBAs) using a single iron-source method and systematically investigates the influence of atmospheric components, particularly water and oxygen, on their oxidative decomposition. Our findings demonstrate that the oxidative degradation of PBAs is governed synergistically by moisture and oxygen, with ambient humidity identified as the primary factor determining both the extent and kinetics of their decomposition. Notably, a pure oxygen environment by itself does not trigger material degradation, while oxygen markedly accelerates the decomposition only in the presence of moisture. As a result of the oxidation, enhanced Coulombic interaction between sodium ions and cyano groups induces structural modifications in the lattice framework, driving a phase transformation from monoclinic to cubic symmetry, accompanied by changes in its unit cell volume. Furthermore, in high-humidity environments, atmospheric moisture promotes the gradual deintercalation of sodium ions from the Prussian blue framework, resulting in the conversion of sodium-rich Prussian blue to the sodium-deficient form. Concurrently, an increase in lattice defect density leads to partial structural collapse, inducing the release of free ferrocyanide ions, which may subsequently react with the deintercalated sodium ions to form the sodium ferrocyanide impurity phase. We also find that the preferential decomposition of low-spin iron over high-spin iron within the framework leads to a further reduction in its electrochemical capacity. In contrast, potassium Prussian blue exhibits minimal interaction with water molecules and can effectively repel them through steric hindrance. Therefore, partial substitution of sodium with potassium ions is proposed as a viable strategy to enhance the structural stability of the Prussian blue framework, improve the storage performance of sodium Prussian blue (NaPB), and mitigate water ingress. This work offers fundamental insights into the storage characteristics and oxidative degradation mechanisms of PBAs.

## 1. Introduction

In the field of sodium-ion batteries, Prussian blue and its analogues (PBAs) are among the three primary categories of cathode materials. With advantages such as abundant raw material reserves and low fabrication costs, they have become a research hotspot [1,2,3,4,5,6]. Various synthesis strategies have been employed to prepare low-defect and high-capacity PBAs materials to enhance their electrochemical performance [7,8,9]. However, moisture content has a significant detrimental impact on the structure and performance of PBAs: crystalline water not only occupies the intercalation sites and diffusion pathways of alkali metal ions but may also promote the formation of lattice defects and structural distortions. These adverse effects promote electrochemical side reactions [10,11], thereby severely compromising the storage stability of PBAs. To address this issue, recent studies have attempted to enhance performance through coating and other methods. For instance, Fu et al. focused on the high content of crystalline water and the susceptibility of metal ions to dissolution within the PBAs lattice, employing a liquid-phase method to deposit a physical barrier coating on Fe-PB, thereby reducing the crystalline water content [12]. Shu et al. grew a ZIF-8 layer on the surface of PB particles through an in situ wet chemical approach to shield against atmospheric moisture and inhibit oxidation, thereby significantly extending the material’s storage lifetime [13]. However, the above works mainly focused on improving storage performance and did not thoroughly investigate the intrinsic mechanisms underlying PBA oxidation and decomposition.

Current research on the dehydration of PBAs mainly focuses on the removal of bulk crystalline water [14,15,16], while the influence of atmospheric moisture and other environmental factors on the stability of PBAs has only received limited attention from researchers. The Ojwang team systematically studied the structural evolution and electrochemical performance changes in Prussian White (PW) under different relative humidity (RH) conditions and proposed a two-step degradation mechanism driven by moisture: the first step is the formation of an alkaline environment on the surface of PBAs through the synergy of oxygen and water, and the second step is the destruction of the surface structure of PBAs by this alkaline environment and the initiation of decomposition [17]. In addition, Louis et al. investigated the storage behavior of sodium-rich Na_1.8_MnHCF and partially sodiumized Na_1.3_MnHCF in high humidity environments and found that under these conditions, impurities such as hydroxides and carbonates would form on the surface of PW, and the bulk water absorption would cause the crystal structure to transform from the rhombohedral phase (fully sodiumized) to the monoclinic phase, with the fully sodiumized PW showing a more significant water-absorption effect [18]. However, although existing studies have pointed out that the oxidation process of Fe^2+^ in PBAs depends on the participation of O_2_, they have not clearly differentiated the individual contribution of H_2_O from the synergistic effect of H_2_O and O_2_ in the degradation process. Therefore, to systematically reveal the oxidation decomposition mechanism of PBAs, it is essential to clarify the distinct and combined influences of H_2_O and O_2_ on the oxidative degradation of PBAs.

According to prior literature, the oxidation of Prussian blue (PB) under ambient conditions is facilitated by surface-adsorbed water and oxygen, which collectively generate a locally alkaline microenvironment that can induce surface degradation. In our 300 L pilot-scale synthesis conducted in a stirred-tank reactor, we observed that during real-world storage and transportation, elevated ambient humidity triggers variable degrees of oxidative degradation—leading to batch-to-batch inconsistencies in electrochemical performance and compromising battery quality control. These observations suggest that moisture exerts a pronounced influence on PBAs stability. The role of moisture in PBAs materials include the following key studies: Fu [19] successfully activated low-spin Fe-based PBAs (PB-LS Fe) via inert-gas-assisted thermal dehydration, effectively removing lattice-bound crystalline water. To accelerate the industrial deployment of PBA cathodes, we are therefore developing a mechanistic understanding of how ambient moisture and oxygen drive structural degradation and electrochemical performance decay, thereby critically determining their practical viability in commercial energy storage applications. Wang systematically investigated vacuum dehydration of PBAs at varying temperatures and established quantitative correlations among residual water content, [Fe(CN)_6_] vacancy concentration (increasing from 4% to 27%), and unit-cell volume expansion (up to 23%) [14]. Moreover, this study demonstrates that controlled dehydration modulates the Fe–C bond length and alters the local coordination geometry, thereby activating the redox activity of low-spin Fe centers. In a complementary approach, Zheng [20] developed a one-step chemical pre-sodiation strategy using sodium benzophenone ketyl in anhydrous toluene, where the reagent serves a dual function: it chemically consumes trace water molecules via proton abstraction while simultaneously enabling Na^+^ insertion into the PBA lattice, thus achieving synergistic dehydration and sodiation in a single operation. Despite the extensive literature on PBA dehydration from both our group and others, investigations specifically targeting moisture- and oxygen-induced degradation during long-term material storage have hitherto remained scarce. To accelerate the industrial deployment of PBA cathodes, we are therefore developing a mechanistic understanding of how ambient moisture and oxygen drive structural degradation and electrochemical performance decay, thereby critically determining their practical viability in commercial energy storage applications.

In this study, we systematically investigated the effects of water and oxygen on Prussian blue analogues (PBAs) by precisely regulating atmospheric conditions. We identified the oxidative decomposition products of sodium Prussian blue (NaPB), confirming that Prussian blue undergoes irreversible degradation to form sodium ferrocyanide salts. Additionally, we demonstrated that water plays a pivotal role in initiating decomposition and material degradation. Under 75% relative humidity (RH), oxygen accelerates PBA decomposition via oxidative reactions, indicating that the oxidative degradation process relies on the synergistic interplay between water and oxygen. Specifically, in the presence of water, Prussian blue initiates decomposition, while oxygen oxidizes approximately 11% of the divalent iron species on the material surface, thereby accelerating overall degradation. In contrast, for the potassium Prussian blue (KPB) system, the larger ionic radius of potassium ions confers enhanced antioxidant properties to the material, enabling it to maintain structural stability without decomposition [21,22]. Thus, potassium ion doping can be leveraged to improve the antioxidant capacity of Prussian blue. We further found that NaPB effectively adsorbs water onto its surface, which diminishes the electrochemical activity of low-spin iron in the material. These findings provide critical guidance for the large-scale commercial application of sodium-ion Prussian blue cathode materials.

## 2. Materials and Methods

### 2.1. Synthesis

All chemical reagents were purchased from the Chemical Reagent Co., Ltd., of Sinopharm Group (Shanghai, China). Na_1.98_Fe[Fe(CN)_6_]_0.99_·0.96H_2_O was synthesized by the hydrothermal method. Then, 15 mmol of Na_2_S_2_O_3_·H_2_O and 5 mmol of Na_4_[Fe(CN)_6_]·10H_2_O were dissolved in 50 mL of 0.1 M HCl, and then transferred to a 100 mL polytetrafluoroethylene (PTFE)-lined autoclave and maintained at 95 °C for 24 h. After filtration, the product was vacuum-dried at 80 °C overnight and designated as NaPB.

Meanwhile, K_1.89_Fe[Fe(CN)_6_]·0.18H_2_O was synthesized by the same hydrothermal method. Next, 5 mmol of Na_4_[Fe(CN)_6_]·10H_2_O was replaced with 5 mmol of K_4_[Fe(CN)_6_]·10H_2_O for the hydrothermal synthesis. After filtration, the product was vacuum-dried at 80 °C overnight and designated as KPB.

### 2.2. Material Characterization

XRD patterns were obtained by irradiating with Cu−Kα in the range of 10° to 60° using a Rigaku Miniflex 600 diffractometer (Rigaku Corporation, Tokyo, Japan). The powder data obtained were indexed and refined using GSAS-II software (v5.2.0). The microstructure of the samples was observed using SEM (FEI Apreo S LoVac, Thermo Fisher Scientific, Brno, Czech Republic). FTIR tests were conducted using a Nicolet iS 50 (Thermo Fisher Scientific, Waltham, MA, USA). VSM tests were performed on a LakeShore 7404 (Lake Shore Cryotronics, Inc., Westerville, OH, USA) to verify the magnetic properties of the samples. Raman spectra were obtained using a LabRAM Aramis instrument (HORIBA Jobin Yvon, Paris, France) with a 532 nm excitation wavelength. XPS was tested using a Thermo K-Alpha instrument (HORIBA Jobin Yvon, France), and the samples were prepared in an argon atmosphere. ICP analysis was performed on an ULTIMA 2 (Elementar, Langenselbold, Germany) to determine the Na, K, and Fe ratios of the samples. The ratios of C, N, O, and H were determined using an elemental analyzer (Vario EI Cube). Electron paramagnetic resonance (EPR) spectra were acquired on an EPR200-V3 spectrometer (CIQTEK Co., Ltd., Hefei, China).

### 2.3. Environmental Control

Before conducting the storage research under different environments, the NaPB powder samples were dried at 130 °C under a vacuum for 24 h. The atmospheres of the four storage devices were as follows: (1) Moist aerobic environment: The NaPB powder was placed in a container, and the humidity inside the container was controlled at around 75% using a saturated NaCl solution. The material placed in this environment was named NaPB-H_2_O-O_2_. (2) Moist anaerobic environment: The NaPB powder was placed in a sealed container and the humidity inside the container was controlled at around 75% using a saturated NaCl solution. To ensure an oxygen-free environment was maintained inside the container, we first introduced argon gas for 1 h, and then introduced argon gas at a flow rate of 10 mL/min for 1 h every 24 h. To ensure no residual oxygen remained, iron powder was added as an antioxidant. The iron powder turned red upon oxidation, but no color change occurred within 90 days, indicating that no external oxygen entered. The material placed in this environment was named the NaPB-H_2_O group. (3) Anhydrous hyperoxic environment: The NaPB powder was placed in a sealed container with a large amount of desiccant silica gel. We first introduced argon gas for 1 h and then introduced high-purity oxygen at a flow rate of 10 mL/min for 1 h every 24 h to ensure a pure oxygen atmosphere inside the container. The material placed in this environment was named NaPB-O_2_. During the 90-day placement period, the silica gel in the environment did not change color and the humidity remained unchanged. (4) Anhydrous anaerobic environment: The NaPB material was vacuum-sealed in an aluminum foil bag and placed in a glove box. The water content in the glove box was controlled below 0.1 ppm, and the oxygen content was controlled below 0.5 ppm. The material placed in this environment was named NaPB-Ar. Additionally, KPB was dried using the same method and stored in the same moist aerobic environment as NaPB. The material placed in this environment was named the KPB-H_2_O-O_2_ group (Figure S1).

### 2.4. Electrochemical Measurements

All battery assembly and testing were carried out in an argon-filled glove box. The materials used were all purchased from Canrd Technology Co., Ltd. (Dongguan, China). The battery electrode slurry was prepared by mixing Prussian blue, Ketjen black, and PVDF at a ratio of 8:1:1, and adding an appropriate amount of 1-methyl-2-pyrrolidone (NMP). The mixture was stirred using a high-speed centrifuge (Kejing, Shenzhen, China) at 800 rpm for 3 min and then at 2000 rpm for 5 min. The obtained slurry was coated onto aluminum foil at a thickness of 100 µm and dried in a hot air oven at 60 °C for 2 h. The dried electrodes were punched into 12 mm diameter discs, ensuring an average active material loading of 1–1.2 mg per disc. Battery assembly was performed using 2016-type cells with glass fiber (GF) separators. The electrolyte consisted of 1 M NaPF_6_ and 5 w% FEC dissolved in a mixture of ethylene carbonate and diethyl carbonate (EC/DEC, 1:1 *v*/*v*). Constant current charge/discharge tests were conducted at room temperature within the voltage range of 2.0 to 4.0 V at a rate of 1 C (150 mA/g) using a Neware CT-4008 battery testing system (Neware Technology Limited, Shenzhen, China). Electrochemical impedance spectroscopy (EIS) tests were performed using a CHI 760 e electrochemical workstation (CH Instrument., Shanghai, China) with a frequency range from 100 kHz to 0.01 Hz. The impedance data were transformed into Distribution of Relaxation Times (DRT) using Matlab software (R2025b).

## 3. Results and Discussions

Based on the previous research of our research group, in this study, sodium thiosulfate was introduced into the single-iron-source method for the synthesis of high-crystallinity sodium-rich Prussian blue analogues (PBAs) to successfully prepare high-sodium and low-defect products (denoted as NaPB) [9]. X-ray diffraction (XRD) characterization results showed that NaPB presented a monoclinic crystal system structure, with sharp and high-intensity diffraction peaks in its XRD pattern, indicating that the material has a highly ordered crystal structure. The chemical composition of NaPB was precisely characterized by inductively coupled plasma optical emission spectrometry (ICP) and CHONS elemental analysis, and its molecular formula was determined to be Na_1.98_Fe [Fe (CN)_6_]_0.99_ (Table S1). Thermogravimetric analysis (TGA) tests (Figure S2) indicated that when the temperature was ≤300 °C, the weight loss of NaPB was approximately 13 wt %, which corresponded to the removal of surface-adsorbed water and lattice interstitial water. Combined with the above composition analysis results, its initial chemical composition was further determined to be Na_1.98_Fe [Fe (CN)_6_]_0.99_·0.96H_2_O.

**Figure 1:**
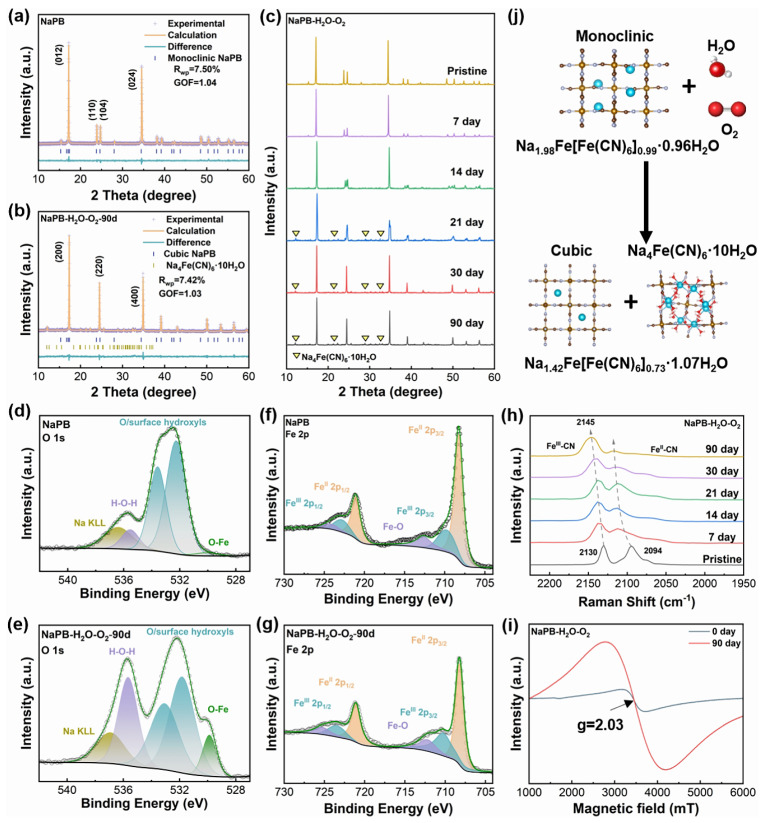
(**a**,**b**) Rietveld refined XRD patterns of NaPB and NaPB-H_2_O-O_2_-90d samples (**c**) XRD patterns of NaPB-H_2_O-O_2_ after different storage durations. (**d**–**g**) O 1s and Fe 2p XPS spectra of the as-prepared NaPB and NaPB-H_2_O-O_2_-90d. (**h**) Raman spectra of NaPB-H_2_O-O_2_. (**i**) EPR spectra of NaPB and NaPB-H_2_O-O_2_-90d. (**j**) Schematic illustration of the oxidation and decomposition process of NaPB.

To verify the air stability of Prussian blue material, it was placed in an air environment for 90 days. The results showed that the material underwent significant color changes (Figure S3a), from light blue to black, and the corresponding 1 C charge–discharge curves of the battery changed. The first discharge platform showed attenuation, and the specific capacity decreased from 133 mAh·g^−1^ to 84 mAh·g^−1^ (Figure S3b,c). Scanning electron microscopy (SEM) images showed (Figure S4a,b) that the surface of freshly prepared NaPB material was smooth and flat; in contrast, the surface of NaPB-90d became rough. The results of thermogravimetric analysis (TGA) indicated that the weight loss of the NaPB-90d sample at 100 °C was approximately 3 wt%, which corresponded to the removal of adsorbed water during the re-hydration process (Figure S2). The weight loss between 200 °C and 300 °C corresponded to the removal of water within the lattice. In this range, NaPB and NaPB-90d lost 13% and 14% of their weight, respectively. The NaPB-90d sample had higher lattice water content, and the dehydration temperature of NaPB (260 °C) was lower than that of NaPB-90d (300 °C), indicating that water in NaPB was more prone to release due to having a more complete crystal structure and higher sodium content [23,24]. After being placed for a period of time, the crystal structure of NaPB-90d was damaged and became incomplete. To clarify the crystal structure changes in NaPB in a humid environment and further evaluate the stability evolution of the material in a humid environment, NaPB samples were subjected to accelerated aging tests in an environment with a relative humidity of 75% (Figure S5), and samples were taken for characterization on the 7th, 14th, 21st, 30th, and 90th days. X-ray diffraction (XRD) tests and Rietveld structure refinement were conducted on samples at different aging stages. As shown in Figure 1a–c, the XRD pattern of the fresh NaPB sample showed obvious (110) and (104) diffraction peaks of the sodium-rich phase; after exposure to a humid and oxygen-rich environment for 14 days, (220) and (200) cubic phase diffraction peaks appeared in the sample, and the original (012) diffraction peak completely disappeared. This phenomenon indicated that NaPB underwent a phase transformation from the sodium-rich monoclinic phase to the sodium-poor cubic phase during the 14-day aging process, accompanied by the loss of sodium ions. When the aging time was extended to 30 days, only (200), (220), and (400) diffraction peaks appeared in the XRD pattern of the sample, indicating that NaPB had completely transformed into the cubic phase. In addition, the characteristic peaks of sodium ferrocyanide decahydrate gradually appeared during the aging process (Figure 1b and Figure S6); the Rietveld refinement results showed that the molar ratio of sodium ferrocyanide decahydrate to cubic phase low-sodium Prussian blue in the sample exposed to a humid environment for 90 days was approximately 1:10. To confirm whether the precipitated crystals contained sodium ferrocyanide decahydrate, the NaPB-H_2_O-O_2_-90d sample was washed, and the washings were evaporated to dryness to obtain the precipitates on the material surface. XRD analysis revealed the characteristic peaks of sodium ferrocyanide decahydrate (Figure S7). Crystal structure parameter analysis indicated that the space group of freshly synthesized NaPB was P21/n, with lattice constants of a = 10.45 (2) Å, b = 7.51 (1) Å, c = 7.27 (1) Å, α = 90.00°, β = 92.70 (3) °, and γ = 90.00°; while after exposure for 90 days, the space group of the sample changed to Fm-3m, with lattice constants of a = b = c = 10.32 (3) Å, α = β = γ = 90.00°, and the unit cell volume slightly increased. The specific fitting parameters are shown in Table S2. This structural evolution is attributed to the enhanced Coulombic interaction between sodium ions and cyano groups after material oxidation, which triggers lattice framework changes, thereby driving the crystal system to transform from monoclinic to cubic and causing a change in the unit cell volume [25].

To clarify the valence state changes in metal ions and the composition of surface deposits, X-ray photoelectron spectroscopy (XPS) analysis was conducted on fresh synthesized NaPB samples and NaPB samples exposed to air for 90 days (Figure S8). The O 1s spectra were used to characterize the existence state of oxygen-containing species on the surface of the two types of samples (Figure 1d,e). The binding energy data analysis indicated that the characteristic peaks at 536.4 eV, 533.6 eV, and 529.9 eV corresponded to the Na KLL Auger peak, crystalline water peak, and O-Fe bonding peak, respectively; [26] the characteristic peaks at 535.5 eV and 532.2 eV corresponded to surface hydroxyl groups, carbon oxides, nitrogen oxides (NO_x_), and other oxygen-containing species [26]. By comparing the O 1s spectra of the two types of samples, it was observed that the areas of the characteristic peaks at 535.5 eV and 529.9 eV in the NaPB sample exposed to a humid and oxygen-rich environment for 90 days (NaPB-H_2_O-O_2_-90d) significantly increased, indicating that a large amount of hydroxyl groups were adsorbed on the surface of the sample during storage, accompanied by the formation of iron oxides and hydroxides. Additionally, the Fe 2p spectra analysis (Figure 1f,g) further clarified the valence state evolution of iron ions on the material surface and the possible phase composition: the characteristic peaks at 708.6 eV and 721.4 eV corresponded to Fe^2+^ 2p_3/2_ and Fe^2+^ 2p_1/2_, respectively; the characteristic peaks at 710.5 eV and 723.6 eV corresponded to Fe^3+^ 2p_3/2_ and Fe^3+^ 2p_1/2_, respectively; and the characteristic peaks at 713.3 eV and 725.5 eV were attributed to iron oxides or hydroxides [27]. Notably, the molar ratio of Fe^3+^ on the sample surface increased from 25% in the fresh sample to 36% in the sample exposed for 90 days, indicating a significant increase in the proportion of trivalent iron ions, confirming that a significant oxidation reaction occurred on the surface of NaPB during storage (Figure S9).

We further revealed the evolution information of water content and iron ion oxidation state in the material through Fourier transform infrared spectroscopy (FTIR) and Raman spectroscopy tests. The infrared spectrum of the NaPB-H_2_O-O_2_ group (Figure S10) shows that the freshly prepared sample has an O-H stretching vibration peak at 3615 cm^−1^ and a H-O-H bending vibration peak at 1616 cm^−1^, which correspond to the crystalline water existing in the bulk phase [28]. Meanwhile, the characteristic peak at 2066 cm^−1^ confirms the strong Fe^2+^-ν(CN) coordination in the material [29], and the sample exposed for 90 days shows a splitting phenomenon in this region. In addition, a new absorption peak appears at 1637 cm^−1^ in the sample exposed for 90 days, which can be attributed to the interaction between adsorbed water molecules on the material surface, indicating that a large amount of water molecules have been adsorbed on the material surface at this time [17]. The Raman spectrum data of NaPB (Figure 1h) shows that the characteristic peaks at 2130 cm^−1^ and 2094 cm^−1^ correspond to the Eg and A1g vibration modes, respectively, both of which are characteristic vibration peaks of Fe^2+^-CN [30], and the above characteristic peaks show a redshift and relative intensity change trend with the extension of the material’s contact time with the environment, and the characteristic peak at 2145 cm^−1^ represents Fe^3+^-CN. The redshift and relative intensity enhancement of this peak indicate that the average valence state of Fe ions in NaPB increases, suggesting an increase in Fe^3+^ content in the material [31], which is consistent with the XPS results. The electron paramagnetic resonance (EPR) test results (Figure 1i) show that the EPR signal at g = 2.03 is due to the unpaired electrons related to CN^−^ vacancies [32], and the signal intensity increases with the increase in the number of CN^−^ vacancies, and the EPR signal intensity of the NaPB-H_2_O-O_2_ group is significantly higher than that of the fresh sample, indicating that NaPB material will induce more CN^−^ vacancies under the long-term action of air, water and oxygen [33]. Based on the above experiments, we infer the oxidation process on the surface of high-crystallinity Prussian blue as shown in Figure 1j. Water molecules can remove sodium ions from the sodium-rich monoclinic phase Prussian blue and form new defects, and yellow blood sodium salt will also be generated on the Prussian blue surface, which is the main destination of the lost ferricyanide and sodium ions.

To further explore the individual mechanisms of water and oxygen in the oxidation process of NaPB, this study designed three comparative experiments and conducted research using the control variable method (Figure S11), namely: the anhydrous and oxygen-free environment storage experiment (named NaPB-Ar), the humid and oxygen-free environment storage experiment (named NaPB-H_2_O), and the dry and pure oxygen environment storage experiment (named NaPB-O_2_). The experimental device schemes for each group are as follows: For NaPB-O_2_, desiccating silica gel was placed to maintain the humidity of the closed environment at below 1% RH. After placing the sample and the hygrometer, O_2_ was introduced for 1 h every 24 h. For NaPB-H_2_O, saturated salt water was placed to stabilize the humidity of the closed environment at 75% RH. After stabilization, the sample was placed, and Ar was introduced for 1 h every 24 h subsequently.

**Figure 2:**
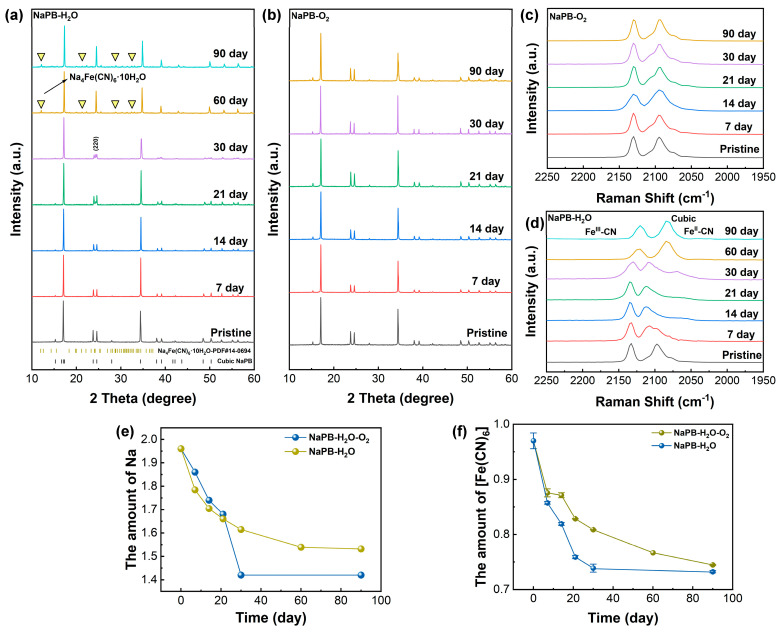
(**a**,**b**) XRD and Raman spectra of NaPB-O_2_ and (**c**,**d**) NaPB-H_2_O samples over time. (**e**,**f**) Time-dependent curves of sodium and ferricyanide content in NaPB-H_2_O-O_2_ and NaPB-H_2_O.

After three months of storage, the XRD and Raman test results of the NaPB-Ar (anhydrous and oxygen-free environment) samples (Figure S12) showed that neither the crystal structure nor the valence state of iron ions had undergone significant changes. This indicates that the optimal storage conditions for Prussian blue materials are vacuum, anhydrous, and oxygen-free or inert atmosphere environments. In contrast, the XRD and Raman data of the NaPB-O_2_ group (dry pure oxygen environment) samples after 90 days of storage (Figure 2a,b) indicated that neither the characteristic peak positions of the XRD pattern nor the Raman spectrum had shifted significantly, suggesting that the crystal structure and valence state of iron ions had not changed significantly either. This indicates that oxygen alone cannot cause the oxidation and decomposition of Prussian blue analogues (PBAs) under anhydrous conditions. To further clarify the impact of water on the stability of NaPB materials, the subsequent analysis focused on the evolution of the crystal structure and valence state of iron ions in the NaPB-H_2_O group (moisture-rich and oxygen-free environment) samples.

After 90 days of storage, the NaPB-H_2_O group (moisture-rich and oxygen-free environment) exhibited a similar performance evolution trend to the NaPB-H_2_O-O_2_ group (moisture-rich and oxygen-rich environment): XRD test results (Figure 2d) showed that the samples in this group also gradually transformed from the monoclinic phase to the cubic phase, and both groups produced sodium ferrocyanide decahydrate. However, the formation rate of sodium ferrocyanide decahydrate in the NaPB-H_2_O group was significantly slower than that in the NaPB-H_2_O-O_2_ group, a difference that could be confirmed by comparing the evolution patterns of the XRD characteristic peaks of the two groups. The NaPB-H_2_O-O_2_ group showed a diffraction peak of yellow blood sodium salt at 21 days, while a distinct diffraction peak of yellow blood sodium salt was observed in the NaPB-H_2_O group around 60 days. The NaPB-H_2_O-O_2_ group samples exhibited a cubic phase characteristic (220) diffraction peak at 14 days of storage, while the NaPB-H_2_O group samples did not show this peak until 30 days. Additionally, the Raman spectroscopy analysis results (Figure 2e) indicated that the characteristic peak at 2130 cm^−1^ corresponding to Fe^2+^ did not shift [30,31], suggesting that there was no oxygen as an oxidant in the system and the valence state of divalent iron in NaPB did not change significantly. However, the original peak at 2093 cm^−1^ shifted significantly to 2112 cm^−1^ [34], which could be attributed to the loss of sodium ions due to long-term contact with water. After 60 days of storage, the XRD data of the NaPB-H_2_O group samples showed that their crystal structure had completely transformed from the monoclinic phase to the cubic phase. In conclusion, it can be inferred that water is the key factor causing the oxidation and decomposition of NaPB during storage, driving the transformation of its crystal structure from the monoclinic phase to the cubic phase with low-sodium and high-defect Prussian blue, and generating sodium ferrocyanide decahydrate impurities. Due to the extremely low oxygen content in the moisture-rich and oxygen-free system, no significant valence state change of divalent iron ions occurred in the material under these conditions. From the thermogravimetric (TG) results (Figure S13), the TG curves of the NaPB-Ar-90d and NaPB-O_2_-90d samples are analogous to that of the pristine NaPB sample, with a 13% weight loss observed between 200 °C and 300 °C, corresponding to the removal of coordinated water. Within this temperature range, the TG curve of the NaPB-H_2_O-90d sample is comparable to that of the NaPB-H_2_O-O_2_-90d sample: a weight loss of approximately 3 wt% occurs at 100 °C, which is attributed to the desorption of adsorbed water from re-humidification of the material, followed by a 14% weight loss between 200 °C and 300 °C. The NaPB-H_2_O-90d sample exhibits higher coordinated water content than the NaPB-Ar-90d and NaPB-O_2_-90d samples, indicating that the lattice structure of NaPB is disrupted in a pure water atmosphere. In contrast, the lattice structure remains intact in argon and pure oxygen atmospheres, as it is not affected by water. The relevant descriptions have been incorporated into the manuscript.

This paper investigates the influence of moisture and oxygen on material degradation during the oxidation process. We find that, under a moist and oxygen-free atmosphere, Prussian blue undergoes proton exchange between hydrated hydrogen ions and sodium ions, yielding alkaline species that corrode the material surface and produce sodium ferrocyanide. This reaction is represented by the following equation.

Na_2_Fe[Fe(CN)_6_] + 2H_2_O → Na(H_3_O)Fe[Fe(CN)_6_] + 2NaOH (1)

Na_2_Fe[Fe(CN)_6_] + 2NaOH → Na_4_Fe(CN)_6_ + Fe(OH)_2_ (2)

Based on the above experimental results, this study proposes a detailed mechanism and evolution process of the interaction between moisture and oxygen in the decomposition of sodium-ion Prussian blue cathode materials: Firstly, the presence of water is the key trigger for the decomposition of PBAs, as water molecules facilitate the gradual de-intercalation of sodium ions from the Prussian blue lattice framework, leading to the transformation of sodium-rich Prussian blue into low-sodium Prussian blue. Simultaneously, the increase in lattice defects causes partial collapse of the framework structure, resulting in the decomposition and release of free ferricyanide ions. The de-intercalated sodium ions then combine with the free ferricyanide ions to form sodium ferricyanide decahydrate. Secondly, the introduction of oxygen significantly accelerates the entire decomposition process. We used ICP and elemental analysis to obtain the sodium and ferricyanide content of the NaPB-H_2_O-O_2_ and NaPB-H_2_O samples (Tables S1 and S3), and the comparison of the decay rates of sodium and ferricyanide content in the two groups of samples confirmed this (Figure 2c,f). We employed ICP and elemental analysis to determine the concentrations of sodium and ferricyanide and found that the decay rates of sodium and ferricyanide content in the NaPB-H_2_O-O_2_ group were significantly faster. After 30 days, the sodium and ferricyanide content of NaPB-H_2_O-O_2_ decreased from 1.98 and 0.99 to 1.42 and 0.72, respectively, while those of NaPB-H_2_O only decreased to 1.64 and 0.81. The article by Ojwang’s team also confirmed that water and oxygen can work together to form an alkaline microenvironment on the surface of Prussian blue materials, thereby accelerating the de-intercalation of sodium ions and the decomposition of lattice defects in sodium-rich Prussian blue [17].

**Figure 3:**
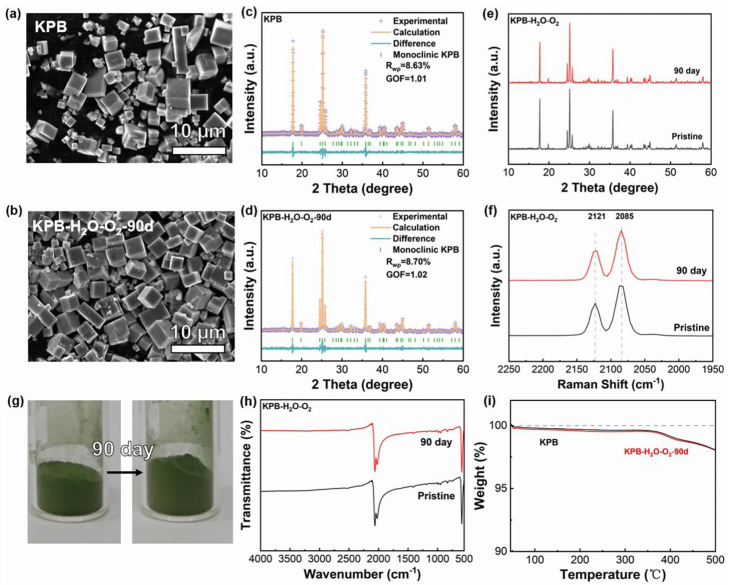
(**a**,**b**) SEM images and (**c**,**d**) Rietveld-refined XRD patterns of KPB samples and KPB-H_2_O-O_2_-90d. (**e**,**f**) XRD and Raman spectra of KPB-H_2_O-O_2_. (**g**) Schematic of degradation in a humid aerobic environment over 90 days. (**h**) FTIR spectrum of KPB-H_2_O–O. (**i**) TGA curves of KPB before and after 90 days.

To explore the influence of alkali metal ions on the antioxidant properties of Prussian blue, this study introduced potassium ion Prussian blue (KPB) and sodium ion Prussian blue (NaPB) for comparative experiments. The KPB material was placed in the same 75%RH air storage environment as the aforementioned NaPB to examine its stability. The results indicated that, in contrast to NaPB, KPB did not show obvious color changes after 90 days of storage (Figure 3g). SEM characterization revealed no significant difference in the microscopic morphology of KPB before and after storage (Figure 3a,b). XRD test results (Figure 3e) showed that the main diffraction peaks of KPB remained sharp and did not shift significantly in the XRD patterns from 0 to 90 days, indicating that no crystal phase transformation occurred in the humid air atmosphere. From the XRD refinement results, the space group of KPB was P21/n, with lattice constants of a = 10.07 (2) Å, b = 7.28 (3) Å, c = 6.94 (2) Å, α = 90.00°, β = 90.19 (2)°, γ = 90.00°, and the space group of KPB-H_2_O-O_2_-90d was P21/n, with lattice constants of a = 10.07 (2) Å, b = 7.28 (2) Å, c = 6.94 (3) Å, α = 90.00°, β = 90.18 (3)°, γ = 90.00°. The cell volumes of the two samples showed no significant difference, indicating that the crystal structure of KPB was more stable than that of NaPB. These results confirmed that potassium ions could enhance the stability of the Prussian blue open framework, thereby protecting the material from oxidation during storage. The reason for this is speculated to be related to the characteristics of potassium ions: potassium ions have a larger radius (about 1.38 Å, while the radius of sodium ions is about 1.02 Å, Figure S11). The larger ionic radius can suppress lattice distortion and reduce water molecule invasion, making the material framework structure more stable [18,31,32]. This is precisely the core reason for the significant difference in air stability between KPB and NaPB and the absence of crystal phase change in KPB in a humid environment (Figure 3c,d). Raman spectroscopy analysis results (Figure 3f) further supported this conclusion: the Fe^2+^-CN characteristic peaks of KPB (2120 cm^−1^, 2082 cm^−1^) did not shift significantly during the 90-day storage process, indicating that the iron ion valence state did not change significantly even in a humid and oxygen-rich environment [35,36], confirming that KPB has excellent antioxidant properties. The infrared spectroscopy comparison results (Figure 3h) showed that the characteristic infrared peaks of KPB in a humid and oxygen-rich environment (2064 cm^−1^, 2024 cm^−1^, 591 cm^−1^) did not change significantly, in sharp contrast to the infrared test results of NaPB, indicating that KPB adsorbed fewer water molecules, while NaPB had a stronger adsorption effect on water. Figure 3i shows that the thermogravimetric curves of the KPB sample placed in a humid and oxygen-rich environment for 90 days and the freshly synthesized sample had no significant difference, indicating that the water content did not change significantly, which is a direct manifestation of the weak interaction between the KPB surface and water molecules. The characteristic of potassium ions stabilizing the Prussian blue framework provides a basis for using K^+^ to replace part of Na^+^ to improve the storage performance of NaPB.

**Figure 4:**
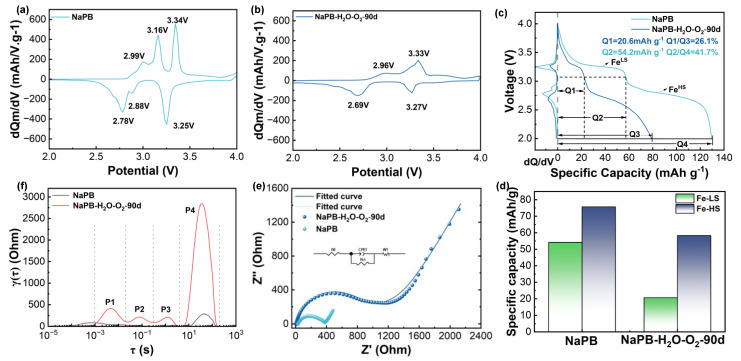
(**a**,**b**) dQ/dV curves of NaPB and NaPB-H_2_O-O_2_-90d. (**c**,**d**) High- and low-spin iron capacity ranges divided from the dQ/dV curves and comparison diagram. (**e**) Electrochemical Impedance Spectroscopy of the NaPB, NaPB-H_2_O-O_2_-90. (**f**) DRT curves.

**Figure 5:**
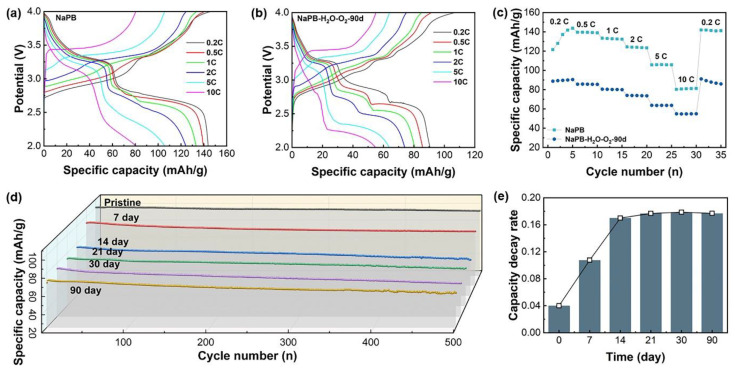
(**a**,**b**) Charge-discharge curves of NaPB and NaPB-H_2_O-O_2_-90d sample at different rates and (**c**) corresponding rates performance. (**d**) Long cycle performance comparisons of NaPB-H_2_O-O_2_ stored for different time at 5 C and (**e**) capacity decay rate.

To explore the intrinsic connection between the oxidation behavior of materials and their electrochemical performance, this study assembled batteries and tested the electrochemical performance of NaPB and NaPB-H_2_O-O_2_-90d samples at a 1 C rate. The focus was on comparing the differential capacity (dQ/dV) curves of fresh NaPB samples and NaPB-H_2_O-O_2_-90d (Figure 4a,b). The results showed that both types of samples exhibited characteristic peaks related to the redox reactions of iron ions in their dQ/dV curves: fresh NaPB samples presented paired redox peaks at 2.99 V/2.78 V, 3.16 V/2.95 V, and 3.34 V/3.25 V. The 2.99 V/2.78 V redox peak corresponded to the redox process of the high-spin iron (Fe-HS)-N_6_ octahedron-20, the 3.34 V/3.25 V peak to the low-spin iron (Fe-LS)-C_6_ octahedron-20, and the 3.16 V/2.95 V peak to the Na 1 site (interstitial), where different redox potentials were observed due to the insertion and extraction of sodium ions at different sites. In contrast, the NaPB-H_2_O-O_2_-90d sample only showed significantly weakened redox peaks at 2.69 V/2.93 V and 3.27 V/3.33 V [37], and its redox potentials were generally lower than those of the fresh NaPB sample. These phenomena indicated that after 90 days of exposure to a humid and oxygen-rich environment, a large number of (Fe-LS)-C_6_ octahedrons in the material became inactive, a change attributed to the oxidation effect of water and oxygen in the air on the material. To precisely quantify the contribution of high-spin iron (Fe-HS) and low-spin iron (Fe-LS) to the total discharge capacity, this study superimposed the dQ/dV curves with the discharge capacity curves (Figure 5c) [38]. This method could clearly separate the discharge capacities attributed to Fe-LS and Fe-HS. The results showed that at a 1 C rate, the discharge capacity contributed by Fe-LS in fresh NaPB samples was 54.2 mAh·g^−1^, accounting for 41.7% of the total capacity, while in NaPB-H_2_O-O_2_-90d samples, the contribution of Fe-LS significantly decreased to 20.6 mAh·g^−1^, accounting for only 26.1%. This indicated that after 90 days of exposure to a humid and oxygen-rich environment, the electrochemical activity of Fe-LS in the material significantly declined, and the contribution of Fe-HS also decreased from 75.2 mAh·g^−1^ to 58.2 mAh·g^−1^ (Figure 4d). This difference suggested that water molecules were more likely to decompose Fe-LS, thereby affecting the redox energy release process of Fe-LS. Additionally, the increased lattice vacancies in the material led to a further decrease in the capacity contributions of both Fe-HS and Fe-LS [39].

To reveal the influence of oxidation degree on the charge transfer kinetics, the charge transfer resistance (Rct) of NaPB and NaPB-H_2_O-O_2_-90d was measured by electrochemical impedance spectroscopy (EIS) [40], and was reported to be 313.4 Ω and 785.7 Ω, respectively (Figure 4e). NaPB-H_2_O-O_2_-90d had a higher charge transfer resistance, which was related to its higher defect content. Figure 4f shows the DRT data of the materials before and after oxidation [23,41]. Four distinct peaks appeared in the oxidized material, while only two peaks were observed in the material before oxidation. Relevant studies have shown that P1, P2, P3, and P4 represent four different polarization processes: I. Electron transfer at the cathode-collector surface layer; II. ion transfer between the cathode and the electrolyte; III. electron transfer within the active material; IV. Na^+^ diffusion within the active material [42]. The cathode–electrolyte interface resistance and the internal charge transfer resistance of the active material in NaPB-H_2_O-O_2_-90d were significantly higher than those of the original material, which might be attributed to the formation of sodium ferrocyanide decahydrate and iron oxide/hydroxide impurity layers on the material’s surface due to oxidation, increasing the cathode–electrolyte interface resistance and the charge transfer resistance.

In addition, this study evaluated the charge–discharge curves of the samples at different rates (Figure 5a,b). The results showed that with the increase in current density, the stable discharge capacities of fresh NaPB samples at 0.2, 0.5, 1, 2, 5, and 10 C rates were 143, 138, 132, 123, 105, and 81 mAh·g^−1^, respectively, while those of NaPB-H_2_O-O_2_-90d samples at the same rates were 90, 85, 80, 73, 63, and 54 mAh·g^−1^ (Figure 5c). Notably, as the current density increased, the discharge platform capacity corresponding to low-spin iron (Fe-LS) of the NaPB-H_2_O-O_2_-90d samples significantly decreased, indicating a marked reduction in the redox activity of Fe-LS. This result further confirmed the adverse effect of air moisture on the Fe-LS activity in Prussian blue analogues (PBAs). Meanwhile, the long-cycle performance of NaPB-H_2_O-O_2_ samples with different storage times was evaluated at a 5 C rate. The relationship between the capacity decay rate and storage time is shown in Figure 5d: the fresh sample exhibited excellent cycling stability over 500 cycles, with an initial discharge capacity of 100.4 mAh·g^−1^ and a remaining capacity of 96.4 mAh·g^−1^ after 500 cycles, resulting in a capacity decay rate of only 4.0%; however, the sample stored in a humid and oxygen-rich environment for 90 days showed significant capacity decay, with the discharge capacity decreasing from 69.5 mAh·g^−1^ to 58.1 mAh·g^−1^ over 500 cycles, leading to a capacity decay rate of 16.5% (Figure 5e). The root cause of the above electrochemical performance differences lies in the loss of sodium ions and the increase in lattice vacancies due to storage in a humid and oxygen-rich environment, which leads to a decrease in Fe-LS activity and a reduction in crystallinity, ultimately resulting in a decline in the cycling stability of the material.

## 4. Conclusions

In this study, we systematically investigated the effects of water and oxygen on PBAs by precisely controlling atmospheric conditions, identified the oxidation decomposition products of NaPB, and demonstrated that water plays a critical role in triggering decomposition and material degradation. Under humidity conditions below 75% RH, oxygen accelerates the decomposition of PBAs through oxidation, indicating that the oxidative degradation of PBAs requires the synergistic action of both water and oxygen. In the presence of water, Prussian blue initiates decomposition, while oxygen concurrently oxidizes approximately 11% of the divalent iron species on the material surface. Oxygen plays a role in accelerating decomposition in this process. The initial capacity of the NaPB-H_2_O-O_2_-90d at 0.2 C decreased from 143 mAh·g^−1^ to 90 mAh·g^−1^, and in the 5 C long cycle, the capacity attenuation rate increased from 4.0% to 16.5% after 500 cycles. For the KPB system, due to the larger ionic radius of potassium ions, Prussian blue exhibits enhanced oxidation resistance and remains stable without decomposition. Additionally, NaPB can effectively adsorb water onto its surface, thereby reducing the Fe-LS activities of the material. This holds significant guidance for the large-scale commercial application of sodium-ion Prussian blue. The research also finds that potassium Prussian blue (KPB) exhibits superior oxidation resistance and structural stability because the larger ionic radius of potassium ions (K^+^) effectively repels water molecules through steric hindrance. This provides a clear strategic basis for improving the storage performance of cathode materials by cation regulation, such as partial potassium substitution.

## Data Availability

The original contributions presented in this study are included in the article/Supplementary Materials. Further inquiries can be directed to the corresponding authors.

## Supplementary Materials

The following supporting information can be downloaded at: https://www.mdpi.com/article/10.3390/ma19142967/s1, Figure S1. Schematic diagram of experimental procedures. Figure S2. TGA image of the initial NaPB and NaPB-H_2_O-O_2_-90d. Figure S3. (a) Color change of NaPB before and after being exposed to air for 90 days. (b) Initial charge–discharge curves at 1C of the initial NaPB sample and NaPB-90d. (c) Capacity plots. Figure S4. (a,b) SEM images of NaPB before and after being exposed to air for 90 days. Figure S5. Experimental setup of NaPB-H_2_O-O_2_. Figure S6. Partial magnification of XRD 10–15° of NaPB-H_2_O-O_2_. Figure S7 XRD pattern of the dried wash solution residue. Figure S8. Full XPS spectra of the initial NaPB sample and NaPB-H_2_O-O_2_-90d. Figure S9. The proportion of Fe^2+^ and Fe^3+^ in the initial NaPB sample and NaPB-H_2_O-O_2_-90d obtained from XPS results. Figure S10. FTIR of NaPB-H_2_O-O_2_. Figure S11. Schematic diagrams of the experimental setups for NaPB-Ar, NaPB-O_2_, and NaPB-H_2_O. Figure S12. (a) XRD pattern and (b) Raman spectrum of NaPB-Ar. Figure S13. The thermogravimetric curves of NaPB-H_2_O-O_2_-90d, NaPB-H_2_O-90d, NaPB-Ar-90d, and NaPB-O_2_-90d. Table S1. Elemental analyzer and ICP analysis results of (a) NaPB-H_2_O-O_2_ and (b) KPB. Table S2. The crystal parameter of (a) NaPB and (b) NaPB-H_2_O-O_2_-90d. Table S3. Elemental analyzer and ICP analysis results of NaPB-H_2_O.
