# Supplementary material for: Investigation into the Storage-Induced Oxidation Mechanism of Prussian Blue Analogues
Source: Materials (Basel). 2026 Jul 9;19(14):2967. doi: 10.3390/ma19142967 (PMC13413233; doi:10.3390/ma19142967)
Supplement: Supplementary file 1 [file materials-19-02967-s001.zip › materials-4370745-supplementary.pdf]

Table S1. Elemental analyzer and ICP analysis results of (a) NaPB-H_2O-O_2 and (b) KPB.

(a) NaPB-H_2O-O_2.

Sample	C	N	O	H	Na	Fe
Pristine	20.12%	23.12%	5.41%	0.71%	13.94%	33.99%
7 day	20.32%	23.74%	5.60%	0.79%	13.25%	35.14%
14 day	20.32%	23.31%	5.81%	0.77%	12.53%	35.28%
21 day	19.67%	22.89%	6.02%	0.81%	12.37%	35.73%
30 day	19.96%	23.25%	5.80%	0.75%	10.95%	37.25%
90 day	19.94%	23.24%	5.62%	0.70%	10.97%	37.98%

All the elements account for mass percentage.

(b) KPB

Sample	C	N	O	H	K	Fe
KPB	21.00%	24.67%	1.02%	0.13%	20.93%	31.73%

All the elements account for mass percentage.

Table S2. The crystal parameter of (a) NaPB and (b) NaPB-H_2O-O_2-90d**.**

(a) NaPB.

Atom	x	y	z	Occupation	U
Fe1	0.0000	0.5000	0.0000	1.0000	0.0028
Fe2	0.0000	0.0000	0.0000	1.0000	0.0063
C1	0.0005	0.3200	0.3270	1.0000	0.0000
C2	0.1724	0.5160	0.5094	1.0000	0.0180
C3	0.0089	0.6766	0.3151	1.0000	0.0208
N1	0.0029	0.2080	0.2106	1.0000	0.0086
N2	0.2924	0.5043	0.4881	1.0000	0.0009
N3	0.0110	0.7965	0.2187	1.0000	0.0000
Na1	0.2460	0.5884	0.0642	1.0000	0.0107

(b) NaPB-H_2O-O_2-90d.

Atom	x	y	z	Occupation	U
Fe1	0.0000	0.0000	0.0000	0.8678	0.0194

Fe2	0.5000	0.0000	0.0000	0.8651	0.0263
C1	0.1894	0.0000	0.0000	1.0000	0.1717
N1	0.3181	0.0000	0.0000	0.9020	0.0389
Na5	0.2500	0.2500	0.2500	0.5700	0.1860

Table S3. Elemental analyzer and ICP analysis results of NaPB-H_2O.

Sample	C	N	O	H	Na	Fe
Pristine	20.12%	23.12%	5.41%	0.71%	13.94%	33.99%
7 day	19.91%	23.27%	5.83%	0.73%	13.58%	36.97%
14 day	19.84%	23.27%	5.44%	0.69%	13.19%	37.72%
21 day	20.08%	23.47%	5.21%	0.66%	12.83%	37.84%
30 day	19.90%	23.29%	5.53%	0.70%	12.69%	38.28%
60 day	20.18%	23.76%	5.80%	0.73%	11.80%	37.35%
90 day	20.17%	23.48%	6.53%	0.84%	11.71%	37.31%

All the elements account for mass percentage.

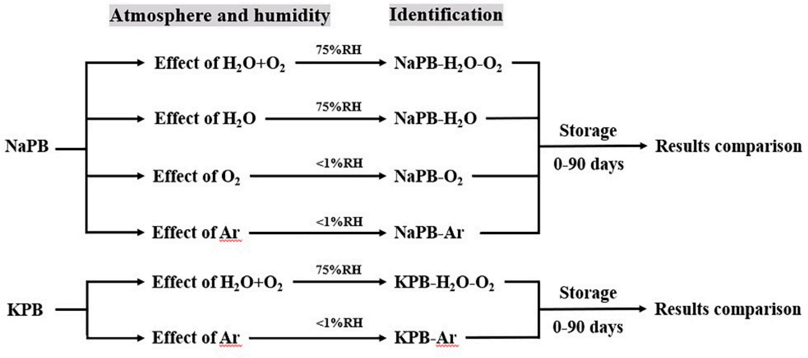

Figure S1. Schematic diagram of experimental procedures.

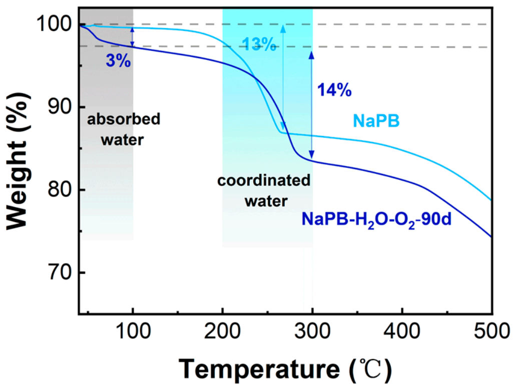

Figure S2. TGA image of the initial NaPB and NaPB-H_2O-O_2-90d.

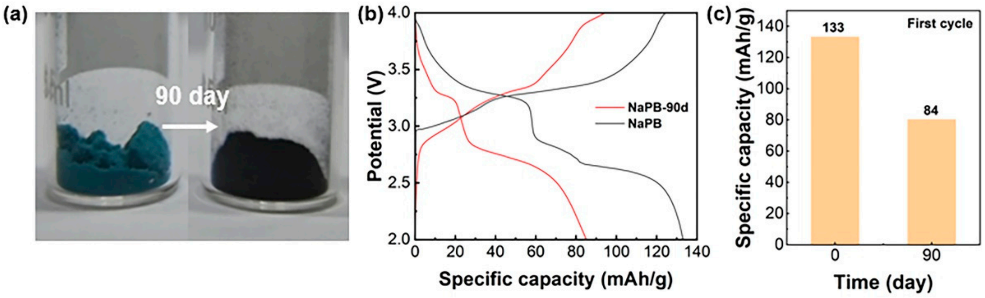

Figure S3. (a) Color change of NaPB before and after being exposed to air for 90 days. (b) Initial charge–discharge curves at 1C of the initial NaPB sample and NaPB-90d. (c) Capacity plots.

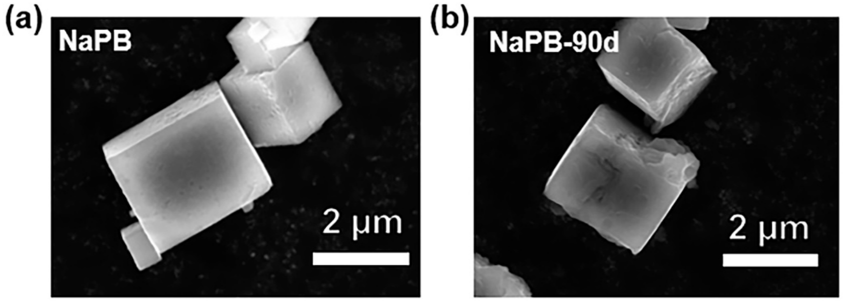

Figure S4. (a,b) SEM images of NaPB before and after being exposed to air for 90 days.

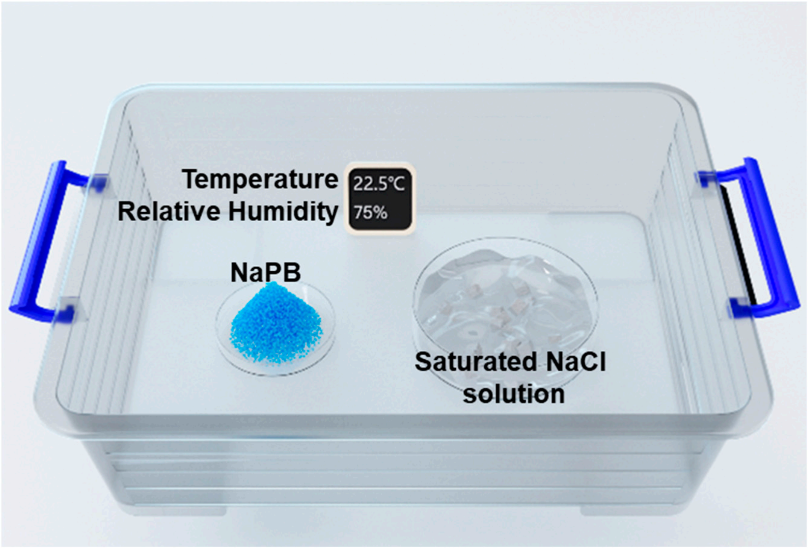

Figure S5. Experimental setup of NaPB-H_2O-O_2.

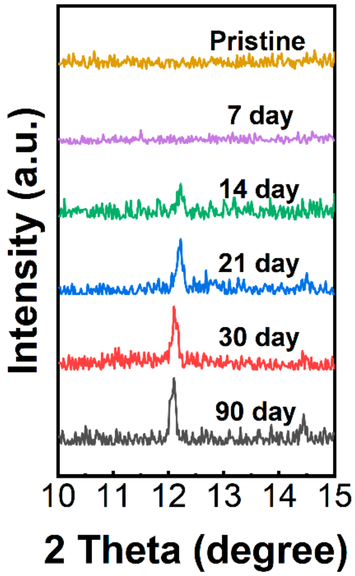

Figure S6. Partial magnification of XRD 10–15° of NaPB-H_2O-O_2.

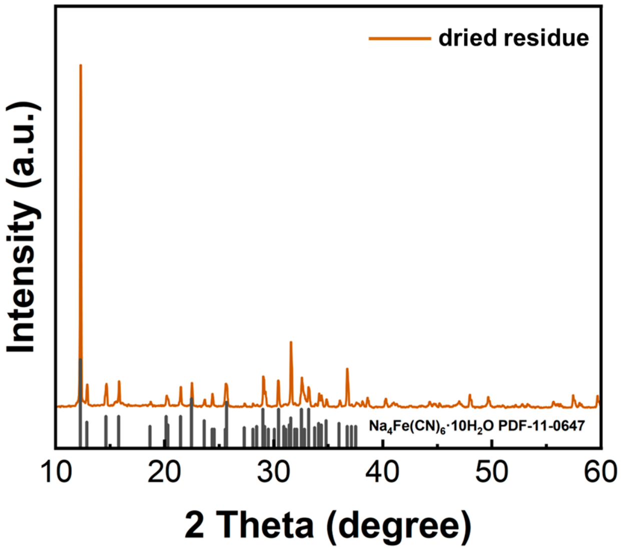

Figure S7 XRD pattern of the dried wash solution residue.

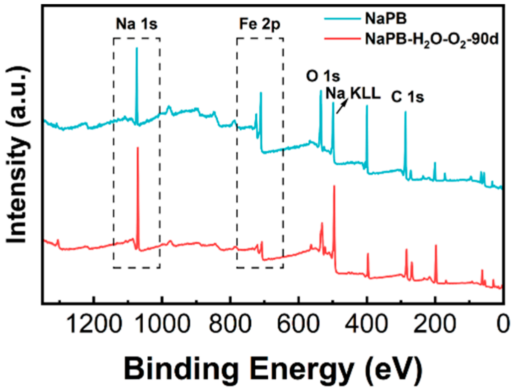

Figure S8. Full XPS spectra of the initial NaPB sample and NaPB-H_2O-O_2-90d.

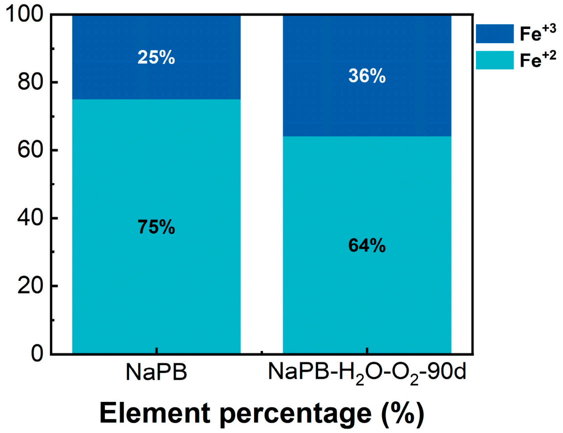

Figure S9. The proportion of Fe^{2+} and Fe^{3+} in the initial NaPB sample and NaPB-H_2O-O_2-90d obtained from XPS results.

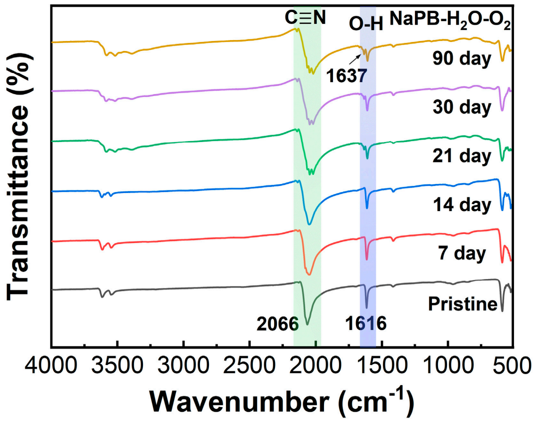

Figure S10. FTIR of NaPB-H_2O-O_2.

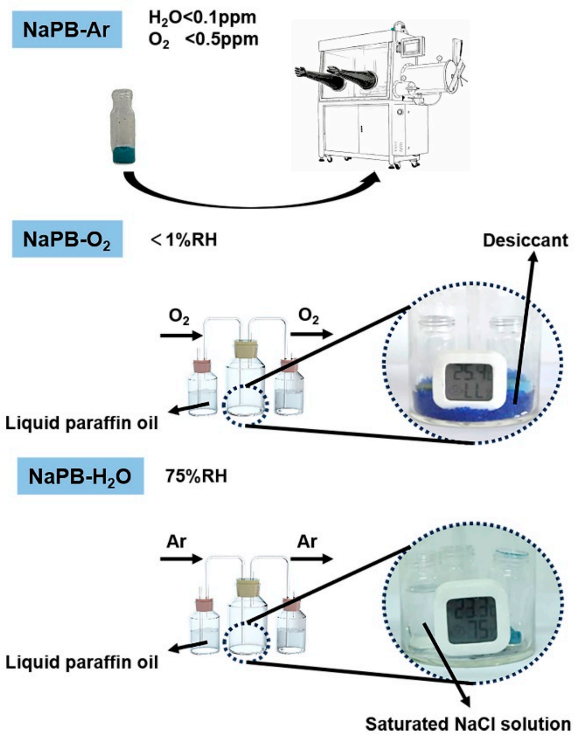

Figure S11. Schematic diagrams of the experimental setups for NaPB-Ar, NaPB-O_2, and NaPB-H_2O.

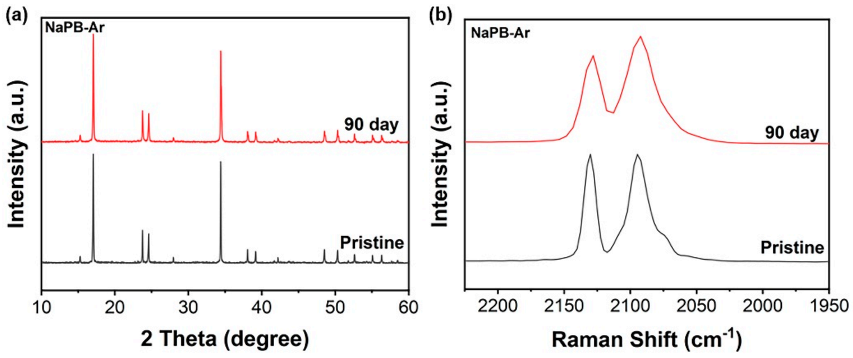

Figure S12. (a) XRD pattern and (b) Raman spectrum of NaPB-Ar.

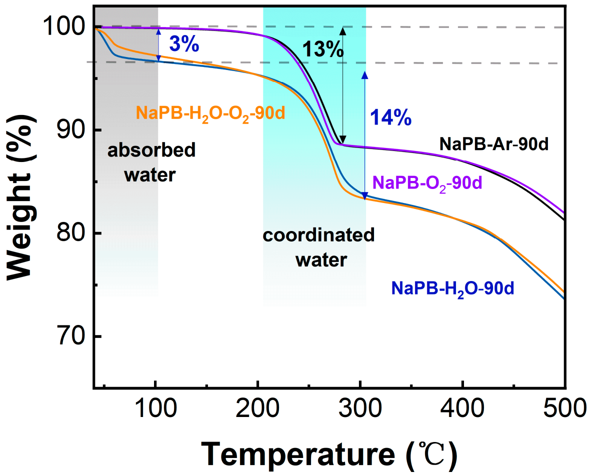

Figure S13 The thermogravimetric curves of NaPB-H_2O-O_2-90d, NaPB-H_2O-90d, NaPB-Ar-90d, and NaPB-O_2-90d.
